# Complete Genome Sequences of Novel Anelloviruses from Laboratory Rats

**DOI:** 10.1128/genomeA.01262-14

**Published:** 2015-02-05

**Authors:** Shoko Nishiyama, Bernadette M. Dutia, Colin P. Sharp

**Affiliations:** Division of Infection and Immunity, The Roslin Institute and Royal (Dick) School of Veterinary Sciences, University of Edinburgh, Edinburgh, United Kingdom

## Abstract

Anelloviruses are nonenveloped single-stranded DNA viruses infecting a wide range of mammals. We report three complete genomes of novel anelloviruses detected in laboratory rats. Phylogenetic analysis demonstrates that these viruses are related to but distinct from recently described rodent Torque teno viruses (RoTTVs) found in wild rodent species.

## GENOME ANNOUNCEMENT

The prototype anellovirus, Torque teno virus (TTV), was described in 1997 in a Japanese patient with posttransfusion hepatitis of unknown etiology (1). While further research has demonstrated the widespread nature and high genetic variability of this viral family (2, 3), their possible role in disease has yet to be fully elucidated. Anellovirus infections have also been observed in a wide variety of wild and domesticated mammalian species (4–13). We recently described infections with two distinct species of rodent anelloviruses (RoTTV1 and RoTTV2) that were present in populations of wild rodents in the United Kingdom, including wood mice (Apodemus sylvaticus), field voles (Microtus agrestis), and bank voles (Myodes glareolus) (14). These viruses were notably absent in house mice (Mus musculus) in both wild and laboratory settings.

In the present study, we used the previously described PanTTV PCR assay (14) to screen 10 laboratory rats (Rattus norvegicus) from 2 separate groups, group A (*n* = 4) and group B (*n* = 6). All 10 rats were found to be anellovirus positive. Sequencing of the cloned PCR amplicons revealed three distinct sequences. The first was present in three members of group A, the second in a single individual from group A, and the third in all members of group B. No evidence of mixed infections was seen. Complete viral DNA was amplified using the previously described technique of rolling circle amplification followed by PCR with inverted primers based on the screening amplicon sequence (14). Representative amplicons from each of the three PCRs were cloned and sequenced by primer walking.

The viral genomes RN_2_Se15, RN_5_Se5, and RN_8_Se11 are 2,572, 2,573, and 2,572 nucleotides (nt) in length, respectively, and sequences share greater than 90% nt identity across the whole genome. All three sequences show a genomic organization consistent with other anelloviruses possessing unidirectional overlapping open reading frames (ORFs). Two of these ORFs were identifiable as ORF1 and ORF2 (nt 273 to 1961 and nt 112 to 351, respectively) based on sequence homology with other anelloviruses. A third predicted ORF (nt 1615–1911) was present in all genomes but showed no homology to any known viral proteins by NCBI BLAST analysis. Pylogenetic analysis of the partial ORF1 sequences (equivalent to the region described in reference 14) showed these viruses are most closely related to members of the proposed RoTTV2 species, despite having amino acid sequence distances across the entire ORF1 sequence of 56.1% to 57.4% relative to these viruses.

This is the first description of prevalent anellovirus infection in commonly used laboratory rodents. The effects of these infections on other experimental procedures, including coinfections, remains to be determined.

### Nucleotide sequence accession numbers.

The complete genome sequences of RN2_Se15, RN_5_SE5 and RN_8_SE11 were deposited in GenBank under the accession numbers KM668486, KM668485, and KM609325, respectively.
